# Person-Centered Web-Based Mobile Health System (Symptoms) for Reporting Symptoms in COVID-19 Vaccinated Individuals: Observational Study of System, Users, and Symptoms

**DOI:** 10.2196/57514

**Published:** 2024-10-30

**Authors:** Emelie Gustafson Hedov, Fredrik Nyberg, Stefan Gustafsson, Huiqi Li, Magnus Gisslén, Johan Sundström

**Affiliations:** 1 Department of Medical Sciences Uppsala University Uppsala Sweden; 2 School of Public Health and Community Medicine, Institute of Medicine Sahlgrenska Academy University of Gothenburg Gothenburg Sweden; 3 Department of Infectious Diseases, Institute of Biomedicine Sahlgrenska Academy University of Gothenburg Gothenburg Sweden; 4 Region Västra Götaland, Department of Infectious Diseases Sahlgrenska University Hospital Gothenburg Sweden; 5 Public Health Agency of Sweden Solna Sweden; 6 The George Institute for Global Health University of New South Wales Sydney Australia

**Keywords:** mHealth, mobile health, patient-reported outcomes, apps, COVID-19, vaccination side effects, web-based symptom reporting

## Abstract

**Background:**

The full spectrum of side effects from COVID-19 vaccinations and infections, including milder symptoms or health effects that do not lead to health care visits, remains unknown. Person-centered self-reporting of symptoms may offer a solution. Monitoring patient-reported outcomes over time will vary in importance for different patients. Individuals have unique needs and preferences, in terms of both communication methods and how the collected information is used to support care.

**Objective:**

This study aims to describe how Symptoms, a system for person-centered self-reporting of symptoms and health-related quality of life, was utilized in investigating COVID-19 vaccine side effects. We illustrate this by presenting data from the Symptoms system in newly vaccinated individuals from the RECOVAC (Register-based large-scale national population study to monitor COVID-19 vaccination effectiveness and safety) study.

**Methods:**

During the COVID-19 pandemic, newly vaccinated individuals were identified as the ideal population to query for milder symptoms related to COVID-19 vaccinations and infections. To this end, we used posters in observation areas at 150 vaccination sites across the Västra Götaland region of Sweden, inviting newly vaccinated individuals to use a novel digital system, Symptoms. In the Symptoms system, users can track their symptoms, functioning, and quality of life as often as they wish, using evidence-based patient-reported outcome measures and short numeric rating scales. These scales cover a prespecified list of symptoms based on common COVID-19 symptoms and previously reported vaccine side effects. Participants could also use numeric rating scales for self-defined symptoms if their symptom was not included on the prespecified list.

**Results:**

A total of 731 people created user accounts and consented to share data for research between July 21, 2021, and September 27, 2022. The majority of users were female (444/731, 60.7%), with a median age of 38 (IQR 30-47) years. Most participants (498/702, 70.9%) did not report any of the comorbidities included in the questionnaire. Of the 731 participants, 563 (77.0%) reported experiencing 1 or more symptoms. The most common symptom was pain at the injection site (486/563, 86.3%), followed by fatigue (181/563, 32.1%) and headache (169/563, 30.0%). In total, 143 unique symptoms were reported. Of these, 29 were from the prespecified list, while the remaining 114 (79.7%) were self-defined entries in the symptom field. This suggests that the flexibility of the self-directed system—allowing individuals to decide which symptoms they consider worth tracking—may be an important feature.

**Conclusions:**

Self-reported symptoms in the Symptoms system appeared to align with previously observed post–COVID-19 vaccination symptoms. The system was relatively easy to use and successfully captured broad, longitudinal data. Its person-centered and self-directed design seemed crucial in capturing the full burden of symptoms experienced by users.

## Introduction

In any health care situation, communication between the patient and the health care provider is crucial. Effective information exchange is essential for achieving optimal therapeutic outcomes and overall health care management. Patients are often an underutilized resource in health care; they understand their own health as well as the needs and goals that are important to them [1].

Patients’ accurate descriptions of symptoms are important for diagnosis and tracking disease progression [2]. A substantial body of literature has documented disparities in how health care professionals assess and document symptoms compared with how patients experience them [3,4].

Patient-reported outcome measures (PROMs) have been an underutilized resource in health care and research, despite their demonstrated significant impact on life expectancy and quality of life [5-7]. The equivalence between paper and electronic PROMs has long been established [8]. Different modes of eliciting patient-reported data present various challenges, but mobile health (mHealth) technology offers the potential for individuals to report how they are feeling in real-time, reducing reliance on approaches affected by recall bias (eg, the peak-end effect) [9]. Patients often struggle to accurately remember symptom levels retrospectively, highlighting the need for patient-centric tools to report and grade symptoms electronically, ideally at the time symptoms occur [3]. Remembering symptom levels, such as the intensity of pain and fatigue, beyond the past several days can be challenging for patients [10]. Additionally, using a person-centered, patient-directed symptom reporting system can be invaluable for capturing previously unknown treatment effects or side effects. It is important to recognize that monitoring patient-reported outcomes over time will vary in importance for different patients. Individuals have unique needs and preferences, in terms of both communication methods and how the collected information is used to support their care [11]. To address these challenges, the *Symptoms* system was developed.

Vaccines were rapidly developed to combat the COVID-19 pandemic. To more comprehensively capture self-reported side effects and infections, including milder symptoms or other health effects that do not necessarily lead to health care contact, this study—part of the RECOVAC (Register-based large-scale national population study to monitor COVID-19 vaccination effectiveness and safety) study described below—was initiated. In the study, the *Symptoms* (Symptoms Europe AB) system for person-centered symptom reporting was made available to individuals receiving vaccines in a large health care region in Sweden. Here, we describe the initial experiences with the system and provide examples of the data collected.

The main objective of this study is to describe the *Symptoms* system. To achieve this, we will present data on how the system was used to collect self-reported vaccine side effects in the newly vaccinated population within the RECOVAC study. We describe the system’s characteristics and explore how participants engage with its person-centered, self-directed features.

## Methods

### Setting

This study was conducted as part of RECOVAC, a substudy of the Swedish COVID-19 Investigation for Future Insights, a Population Epidemiology Approach using Register Linkage (SCIFI-PEARL) [12] project. Sweden’s high-quality national health and population registers, which can be linked with a high degree of accuracy, provide an ideal environment for epidemiological research. The Swedish Personal Identity Number (PIN; personnummer) is uniquely assigned to each individual, enabling the compilation and linkage of data from various registers to the same individuals with relative ease and exceptionally high accuracy [13]. In RECOVAC/SCIFI-PEARL, data from the National Vaccination Register, the national database of notifiable diseases (SmiNet), the National Patient Register, the Cause of Death Register, several national quality registers, and primary care registers in the Stockholm and Västra Götaland regions were linked. Additionally, linkage was established with the person-centered symptom reporting system, *Symptoms*, if participants provided consent for the use of *Symptoms* data for this purpose, as described below.

### Ethical Considerations

The study is part of the SCIFI-PEARL project, and ethical approval was obtained from the Swedish Ethical Review Authority (approval number 2020-01800, with subsequent amendments). Informed consent was secured from all participants within the *Symptoms* system, as described in the following paragraphs. The protection of study data privacy and confidentiality is detailed under the section “Handling of Study Data,” while the privacy protection of user data is outlined under the “Development Process of *Symptoms* and Previous Formative Evaluations” section. Study data are pseudonymized, and there were no financial costs or compensation for participants.

### Recruitment of Study Participants

Individuals who had just received a COVID-19 vaccination were identified as the ideal population for this research, partly due to their assumed reduced susceptibility to infection and their potential experience of side effects. Therefore, we invited newly vaccinated individuals to use the novel digital system, *Symptoms*, to track any symptoms and potential side effects from the vaccination.

Vaccination clinics or temporary vaccination sites in the Västra Götaland region were identified, and 176 of these were deemed appropriate and approached through the regional vaccination coordination office. There were no proportional quotas based on sociodemographic variables or vaccine type for the study population; all individuals receiving a vaccination were targeted. The regional vaccination coordination office informed vaccination clinics about the project via email before distributing posters and informational materials.

Posters and flyers were distributed to the vaccination sites in July 2021. Access to 26 locations was not possible (eg, due to locked doors or the absence of vaccinations), or they would not accept the posters. Written instructions were provided to the personnel at the vaccination sites; however, no additional action was required from health care or vaccination staff other than supplying those being vaccinated with the information. This approach was taken to avoid overburdening an already strained organization during the vaccination campaigns. Vaccination points were encouraged to place the posters in the postvaccination waiting area, where all vaccinated individuals were asked to sit and wait for 15 minutes after their vaccination. This was a recommendation for all COVID-19 vaccinations in Sweden at the time, aimed at ensuring immediate care in case of acute postvaccination symptoms. Interested individuals could then access the website, read more, and begin using the application directly, if desired. In January 2022, a second round of posters and flyers was distributed to the vaccination sites.

The posters and flyers provided brief information about the study, along with an easy-to-remember URL and a QR code leading to a landing web page. On this landing page, participants could read the participant information for the study and were asked to provide digital informed consent using BankID, the Swedish secure authentication system uniquely linked to each individual.

The detailed request for participation in the research study was issued within the *Symptoms* system after participants authenticated themselves by logging in with BankID. Using BankID was a prerequisite for participation and served as a technical measure to ensure that each participant could create only 1 account. Once informed consent was provided, participants could access the web-based *Symptoms* system, which is compatible with smartphones, tablets, and computers as needed for the study.

### Symptom-Reporting Tool

In the *Symptoms* system, users can chart their symptoms, functionality, and quality of life as often as they wish, utilizing evidence-based PROMs and short numeric rating scales (NRSs) for various symptoms. Additionally, users can write free-text notes about anything they choose, including medication and self-care. The system is person-centered, viewing the individual as an expert on themselves and their health, and focusing on the whole person rather than solely on diseases. For users consenting to this study, common symptoms of COVID-19 and vaccine side effects were available for selection from a list. However, users could also write in their own symptoms as free text and provide an NRS rating for them. Users could also draw localized symptoms on 3D manikins. Other PROMs available for users’ benefit within the system, accessible as needed, included RAND-36 [14], Välmåendeskalan [15], Montgomery-Asberg Depression Rating Scale [16], and Karolinska Exhaustion Disorder Scale [17]. The use of BankID ensures participant identity, and because it is closely linked to personal identification, it also enables connection with other data sources.

### Development Process of Symptoms and Previous Formative Evaluations

The *Symptoms* system is developed using a modern architecture. The development approach follows a software life cycle model consisting of 5 main stages: planning, development, testing, production, and maintenance. The system’s backend is built with .NET, while the front end uses Vue (Evan You and the Core Team). Both the front end and backend are operated as Docker containers. As the system operates in a container environment, multiple instances of each subsystem can run in parallel, reducing the risk of interruptions. The *Symptoms* system separates user-generated data from user-identifying data, storing them in different databases to minimize the risk of individual user identification in the event of a data breach. Additionally, data in transit are encrypted using Transport Layer Security (HTTPS). On users’ devices, data are encrypted locally using an encryption key stored in the *Symptoms* database. If a user loses their device, simply logging in and disabling the active session on the lost device will secure the data, rendering it impossible to decrypt. The system is developed according to best practices, and data are stored in data centers that meet stringent requirements for connectivity, backup power, cooling, and physical security. All code libraries and other software used in the development process must be well-documented, widely used, and free of known security vulnerabilities that could affect the system’s operation. Additionally, code libraries and frameworks should be regularly reviewed to update to newer versions and address any potential security flaws.

The *Symptoms* system has been developed iteratively in collaboration with patient organizations in Sweden. A group of lead user patients evaluated the system during a project in 2019. Lead user patients are individuals who wish to participate in their own care and engage in a constructive, knowledge-based manner [18,19]. Additionally, members from various patient advocacy organizations, primarily those representing individuals with fibromyalgia and youth with cancer (*Ung Cancer*), contributed during the early development cycles, which involved focus groups.

A feasibility study of an early version of the *Symptoms* system was conducted during the fall of 2017 [20]. This investigation, part of a master’s thesis, was carried out among patients seeking emergency department care for pain at Uppsala University Hospital. The inclusion criteria were as follows: participants had to be over 18 years of age, Swedish-speaking, and experiencing pain. Patients with unstable medical conditions, cognitive impairments, severely impaired vision, or without access to a smartphone were excluded. The study continued until 50 participants completed the trial. After completing their pain reports, participants were asked to fill out a digital evaluation questionnaire. The survey included a Swedish translation of the System Usability Scale, a well-validated method for measuring usability. Overall, the study indicated that participants found reporting pain using the *Symptoms* system useful, and they noted that the 3D manikin was easy to learn and helped facilitate their pain descriptions. There was also a positive attitude toward reporting symptoms using participants’ own mobile phones. Several patients responded very positively to the *Symptoms* concept, particularly those with long-term pain conditions such as fibromyalgia and endometriosis, who expressed a need for new tools to map their symptoms and communicate them to health care providers. The average System Usability Scale score of 79.5 indicated acceptable usability [21]. Usability was weakly inversely correlated with patient age. Although reported usability was not linked to pain intensity, some users declined participation due to being in too much pain at the time. The feasibility study identified several technical hurdles, notably issues with using the system across different smartphone models and operating systems, particularly in displaying the 3D manikin. These technical challenges were addressed in subsequent development.

Since the feasibility study, the system has undergone several development cycles. In 2020 and 2021, a group of patients from an organization for individuals with edema provided insights through semistructured individual interviews conducted by a usability expert. These interviews aimed to better understand how this patient group interacted with the system, leading to further adaptations. Notable changes during the development process included transitioning to more flexible symptom reporting and improvements in how users navigate and mark symptoms on the 3D manikin.

The prior development of the symptom reporting system enabled rapid implementation of necessary modifications before the study began. In addition to front-end updates tailored to the specific study, a significant backend update was conducted. This involved upgrading the operating environment and ensuring that databases and data were located in Sweden before the commencement of participant recruitment.

For this study’s application, no additional usability testing was conducted.

### Study-Specific Entrance and Adaptation of Symptoms

**Figure 1:**
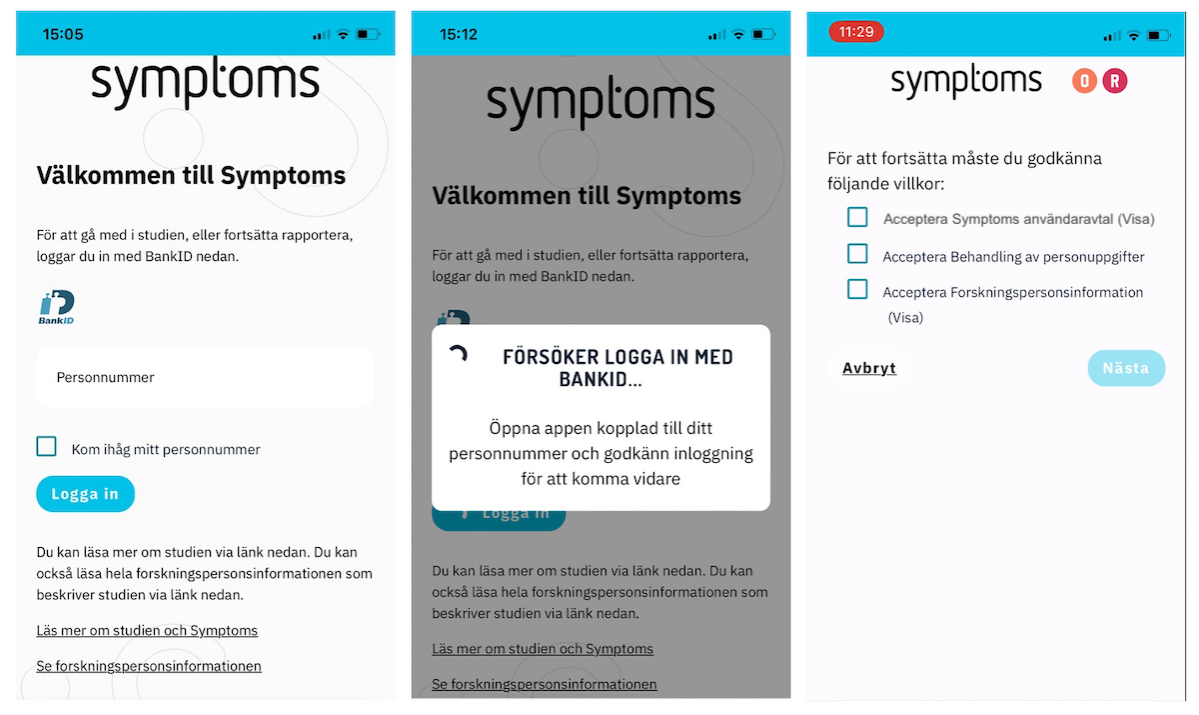
Screenshots from the Symptoms system during recruitment for the study (July 2021): The landing page, login screen waiting for approval by BankID, and screen for approving agreements.

**Figure 2:**
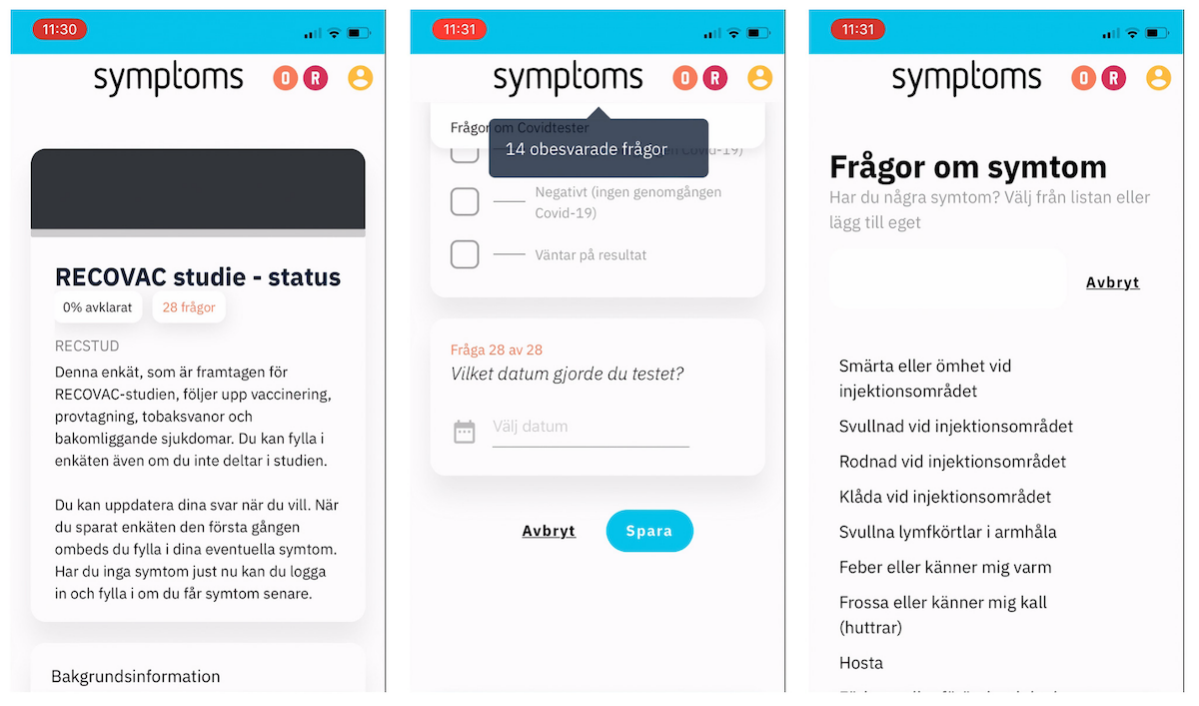
Screenshots from the Symptoms system during the study (July 2021 to September 2022): The start and end of the initial questionnaire and (far right) the subsequent screen for initial symptom reporting, where common symptoms or side effects are listed. The users were also free to type their own symptoms.

**Figure 3:**
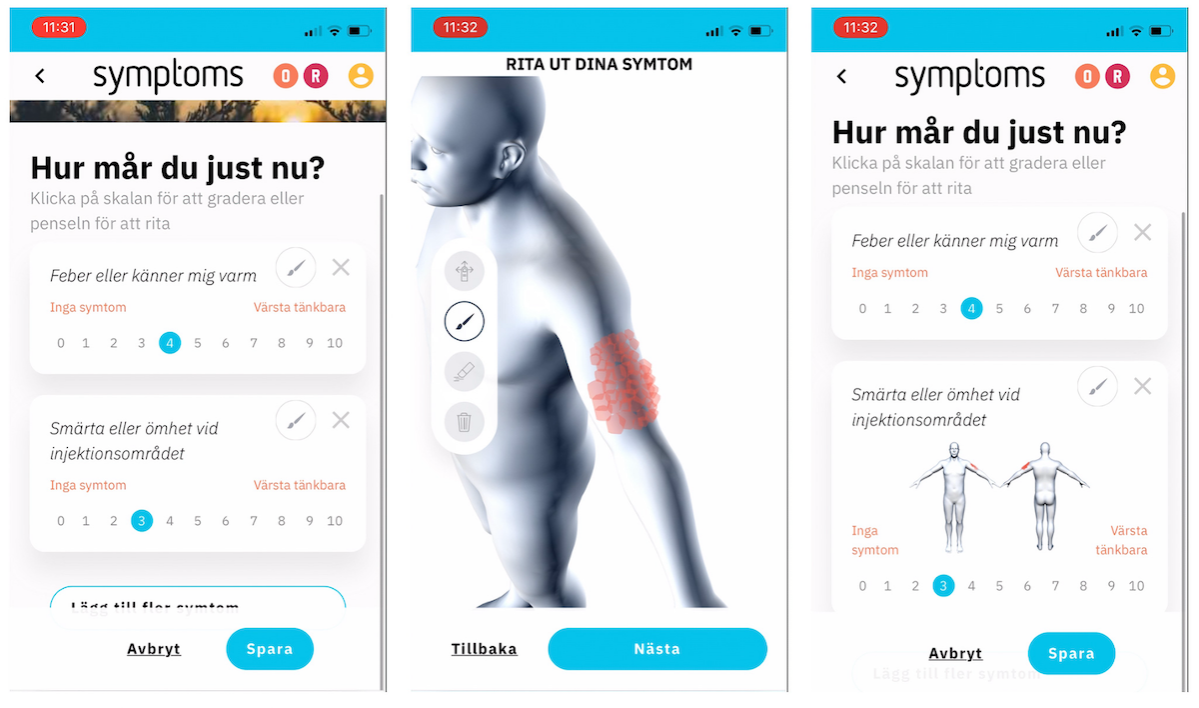
Screenshots from the Symptoms system during the study period (July 2021 to September 2022): Grading of symptoms, drawing of symptoms on the 3D manikin, and grading of symptoms with updated drawing.

**Figure 4:**
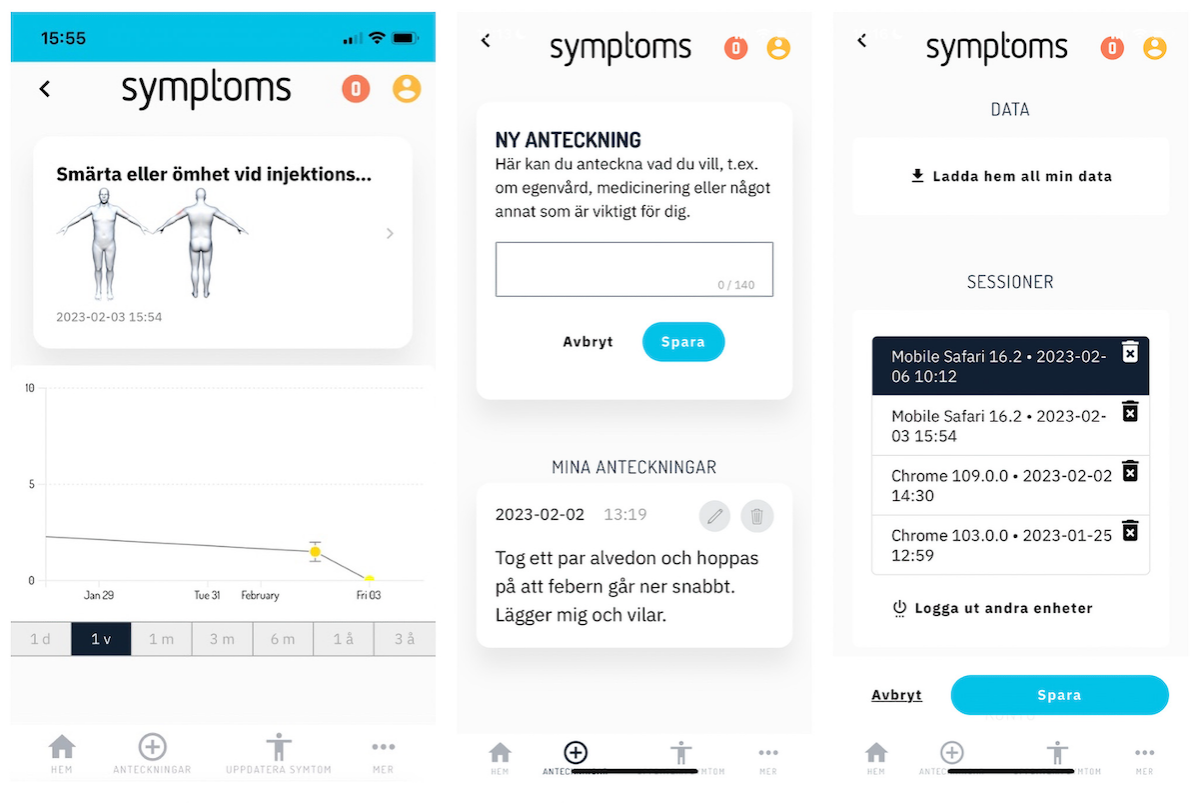
Screenshots from the Symptoms system as it looked during the study period (July 2021 to September 2022): Graph of a symptom with a corresponding drawing, screen for free-text notes, the settings page with options for data export of a person’s own Symptoms data, and active sessions on the same or different devices.

Participants accessed the application by scanning a QR code or typing the web address given in [22]. There, they could read information about the study, access the participant information sheet, and follow links to the *Symptoms* website for more details about the study and answers to frequently asked questions (Figure 1). Translations of the screenshots shown in figures 1-4 are available in Multimedia Appendix 1.

Potential participants had the option to use the *Symptoms* application without enrolling in the study if they chose not to provide study-specific consent; in that case, they would not receive study-specific information within the system. Participants were also informed that they could withdraw their informed consent to share data with the study and still use the self-reporting system. There was no financial cost or compensation for participants.

The symptom-reporting system, *Symptoms*, is continuously updated. Figure 1 illustrates its appearance at the start of recruitment for this study. A short video walkthrough is available in Multimedia Appendix 2.

The self-reported system was adapted so that, after logging in with BankID, users were presented with 3 agreements: a user agreement, a document outlining how personal data would be processed, and a consent form for sharing data with the study. Agreeing to the *User Agreement* and the *Processing of Personal Data* documents was mandatory to create an account. However, sharing data with the research study—outlined in the document that includes statements about voluntary participation and confidentiality—was only required for those who wished to participate in the study (Figure 1).

Users were then prompted to complete a study-specific questionnaire (Figure 2), which took approximately 5 minutes in the initial iteration. Participants were asked to provide background information on their medical history and tobacco use, as well as details about any prior COVID-19 infections by specifying their polymerase chain reaction test results or antigen test results. The final section of the questionnaire focused on vaccinations, where participants were required to provide the date of vaccination and the type of vaccine received. Participants had the option to skip any questions they preferred not to answer while still being able to save their progress in the questionnaire (Figure 2).

After saving the questionnaire, participants were asked if they were experiencing any current symptoms. They were presented with a list of common vaccination side effects and typical COVID-19 symptoms. Additionally, users had the option to report 1 or more symptoms not included in the list by providing a free-text response (Figure 2).

### Selection of Symptoms and Vaccine Side Effects to Provide as Suggestions in the System

The selection of the most common symptoms for COVID-19 to present in the system was based on current literature available at the time of development (December-March 2021). Symptoms such as anosmia (loss of smell), ageusia (loss of taste), and fever or feeling warm [23,24] were included, as they were considered important for distinguishing between individuals with COVID-19 and those without. In addition, multiple studies identified cough, shortness of breath, gastrointestinal symptoms [25,26], as well as nausea, chills [27], and fatigue as important symptoms that could potentially predict the severity of COVID-19 [26,28,29].

The most commonly reported side effects were extracted from the Swedish Medical Products Agency’s reports [30] dated March 17, 2021, for the 3 vaccines in use in Sweden at that time: Vaxzevria (AstraZeneca), Comirnaty (Pfizer-BioNTech), and Spikevax (Moderna). Suspected side effects reported more than 10 times included the following for Comirnaty: fever, fatigue, pain at the injection site, headache, dizziness, numbness, muscle aches, nausea, vomiting, diarrhea, dyspnea, coughing, sore throat, redness of the skin, itchiness, urticaria, heart palpitations, low blood pressure, high blood pressure, runny nose, and herpes zoster. For Vaxzevria, the reported side effects included fever, chills, fatigue, headaches, dizziness, numbness, muscle aches, nausea, vomiting, and heart palpitations. For Spikevax, the side effects included fever, chills, pain at the injection site, headache, muscle aches, and nausea.

### Using Symptoms

Participants could chart their symptoms, functions, and quality of life as often as they liked. The symptoms recorded were rated on an NRS from 0 to 10, where 0=no symptom and 10=worst possible symptom. Users could also utilize evidence-based PROMs provided on the platform. Additionally, users could draw localized symptoms on a 3D manikin (Figure 3). Drawings are linked to a specific symptom by first selecting or typing the symptom and then clicking the brush icon to access the manikin screen. Once in the manikin view, users can turn and spin the manikin using common touch commands such as pinch and zoom, and use the provided menu to switch between drawing, viewing, and erasing drawings. If the user wants to update a previously drawn symptom, for instance, to modify the localization or spread of a symptom, this can be done in the same manner. Users can also view graphs of their symptoms and write free-text notes about anything they choose, including medication and self-care (Figure 4).

Links to a page containing frequently asked questions and information on how to receive support with the *Symptoms* system or the study were available within the system and on the study’s information page.

### Handling of Study Data

Participants have access to their own original data in the *Symptoms* system and can continue to provide new data within the system. A copy of the participants’ data from the self-reporting system was transferred to the National Board of Health and Welfare at specific intervals, where it was collated for the RECOVAC/SCIFI-PEARL study. The data were encrypted and compressed according to the instructions from the National Board of Health and Welfare and sent by registered mail on a USB drive, as specified. Data linkages and pseudonymization of the exported research data are managed in secure environments at the National Board of Health and Welfare and Statistics Sweden. The research database, which includes the symptom data, is housed on a dedicated server at the Centre of Registers in Västra Götaland. Data transfers utilize state-of-the-art encryption, and the project-specific hardware is maintained with high protection levels suitable for sensitive personal data. Protected data storage and infrastructure are maintained and backed up daily.

### Statistical Analyses

Continuous variables are described using medians and IQRs. Categorical variables are presented as numbers and percentages. Participants were asked to select any current or previous comorbid conditions from a list in the questionnaire.

Based on individual self-reported height and weight, BMI was calculated using the following formula: BMI (kg/m^2^)=weight/(height × height).

“Ever smoker” was defined to include both current and former smokers, and the same definition applies to “Ever snuff.” Please see Multimedia Appendix 3 for the initial questionnaire.

## Results

From the start of the study, when posters and flyers were made available at vaccination clinics on July 21, 2021, until the data export on September 27, 2022, a total of 731 individuals created an account and consented to share their data with the study. Demographics from their baseline questionnaire responses are presented in Table 1. The majority of participants were female (444/731, 60.7%), and the median age was 38 (IQR 30-47) years. Most participants reported no comorbidities (498/702, 70.9%), with the most common comorbidity being asthma (92/702, 13.1%), followed by hypertension (54/702, 7.7%).

Among the 731 participants, 556 (76.1%) had previously taken a COVID-19 test. Of these, 163 (29.3%) tested positive.

Nearly all participants (n=726, 99.3%), reported having received their first dose of the vaccine, with a significant majority (586/726, 80.7%) having received Comirnaty. Additionally, 545 (75.4%) reported having taken the second dose, with an even larger proportion (n=454, 83.3%) for Comirnaty.

The usage of the system is reflected in the symptoms reported by participants. Of the 731 participants, 563 (77%) reported experiencing 1 or more symptoms (Table 2). The most frequently reported symptom was pain or tenderness at the injection site (486/563, 86.3%), followed by fatigue (181/563, 32.1%) and headache (169/563, 30.0%). Additionally, muscle pain (160/563, 28.4%) and fever (148/563, 26.3%) were commonly reported, although slightly less so.

**Table 1:** Participant characteristics at the time of reporting the baseline questionnaire in the system^a,b^ among persons newly vaccinated against COVID-19 between July 21, 2021, and September 27, 2022 (N=731).

Characteristics				Values
Age (years)				38 (30-47)
Male sex				287 (39.3)
BMI (kg/m^2^)				25 (22.6-28.3)
Ever smoker				177 (24.2)
Ever snuff				127 (17.4)
**Comorbidities (n=702)^c^**				
	None of the listed			498 (70.9)
	Myocardial infarction			5 (0.7)
	Angina pectoris			3 (0.4)
	Atrial fibrillation			5 (0.7)
	Heart failure			3 (0.4)
	Valve disease			4 (0.6)
	Stroke			2 (0.3)
	Hypertension			54 (7.7)
	Dyslipidemia			32 (4.6)
	Diabetes			9 (1.3)
	Chronic obstructive pulmonary disease			1 (0.1)
	Asthma			92 (13.1)
	Other pulmonary disease			3 (0.4)
	Crohn disease			3 (0.4)
	Autoimmune diseases			21 (3)
	Cancer			24 (3.4)
**Prior COVID-19**				
	**COVID-19 test**			556 (76.1)
		Positive COVID-19 test		163 (29.3)
**Vaccine doses received**				
	**First dose**			726 (99.3)
		First-dose Vaxzevria		23 (3.2)
		First-dose Comirnaty		586 (80.7)
		First-dose Spikevax		117 (16.1)
	**Second dose**			545 (75.4)
		Second-dose Vaxzevria		12 (2.2)
		Second-dose Comirnaty		454 (83.3)
		Second-dose Spikevax		81 (14.9)

^a^Median (IQR) or n (%) are reported. The denominator is not the same for all percentage calculations.

^b^Most questions have been completed but some have missing values, affecting the denominator in percent calculations.

^c^Does not add up to 100% as multiple comorbidities may be reported.

**Table 2:** Number of individuals reporting the 5 most common symptoms, and 5 of the least common symptoms, in the system^a^ among 731 persons newly vaccinated against COVID-19 between July 21, 2021, and Sept 27, 2022.

Characteristics		Values, n (%)
**Has reported symptoms**		563 (77.0)
	Pain or tenderness at the injection site^b^	486 (86.3)
	Fatigue^b^	181 (32.1)
	Headache^b^	169 (30.0)
	Muscle pain^b^	160 (28.4)
	Fever^b^	148 (26.3)
	Delayed and changed menstrual cycle^c^	1 (0.2)
	Hives^c^	1 (0.2)
	Sinus pain^c^	1 (0.2)
	Blood sugar disturbance^c^	1 (0.2)
	Sensitive teeth^c^	1 (0.2)
Has symptom drawing on a manikin		33 (5.9)

^a^Denominators are not the same for all percent calculations. The *has reported symptoms* uses the total number of participants as the denominator, while the individual symptoms use the *has reported symptoms* as the denominator.

^b^Included in the list of symptoms shown to the user.

^c^Symptoms added by the users, not in the list of prespecified symptoms.

**Figure 5:**
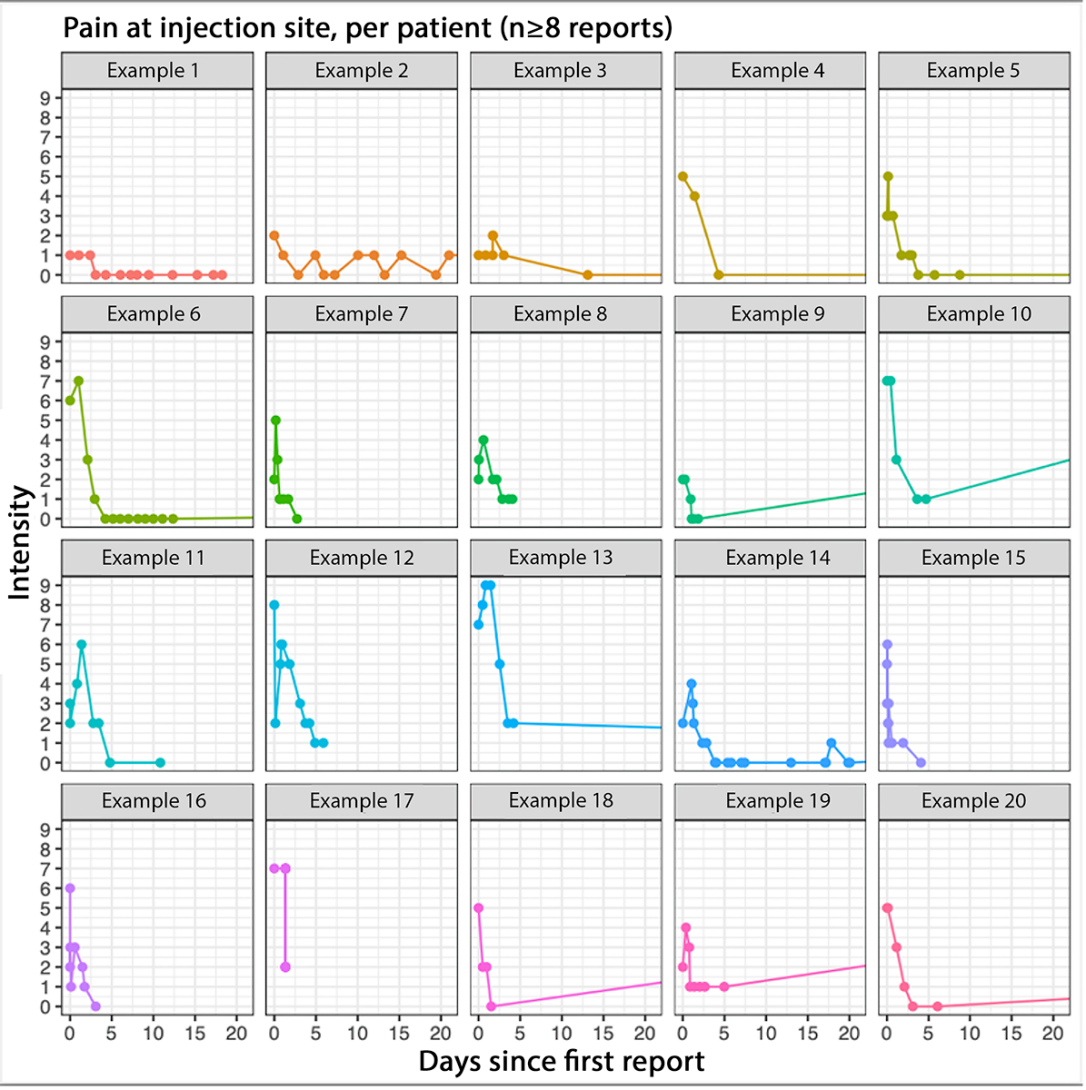
Twenty examples of reported intensity of "pain or tenderness at the injection site" from different individuals over the 20 days since the first report. The figure only shows individuals who reported a symptom at least eight times. Note that the same individual could have reported the symptom multiple times on the same day, and at the same intensity, which will not be evident in the graph. Data from persons newly vaccinated against COVID-19 between July 21, 2021, and September 27, 2022.

Figure 5 presents examples of participants who reported pain at the injection site more than 8 times. It is noteworthy that these examples, which may not be representative of the overall trend in the full population, indicate that many participants reported symptoms frequently in the first few days. Symptoms appeared to be more intense during the initial day(s) following vaccination. Some individuals, such as examples 7, 11, 15, and 16, demonstrated a decrease in symptom intensity over time, eventually reaching 0. However, other participants, such as examples 8, 12, and 17, ceased reporting their symptoms while still indicating an intensity of 1 or higher.

**Figure 6:**
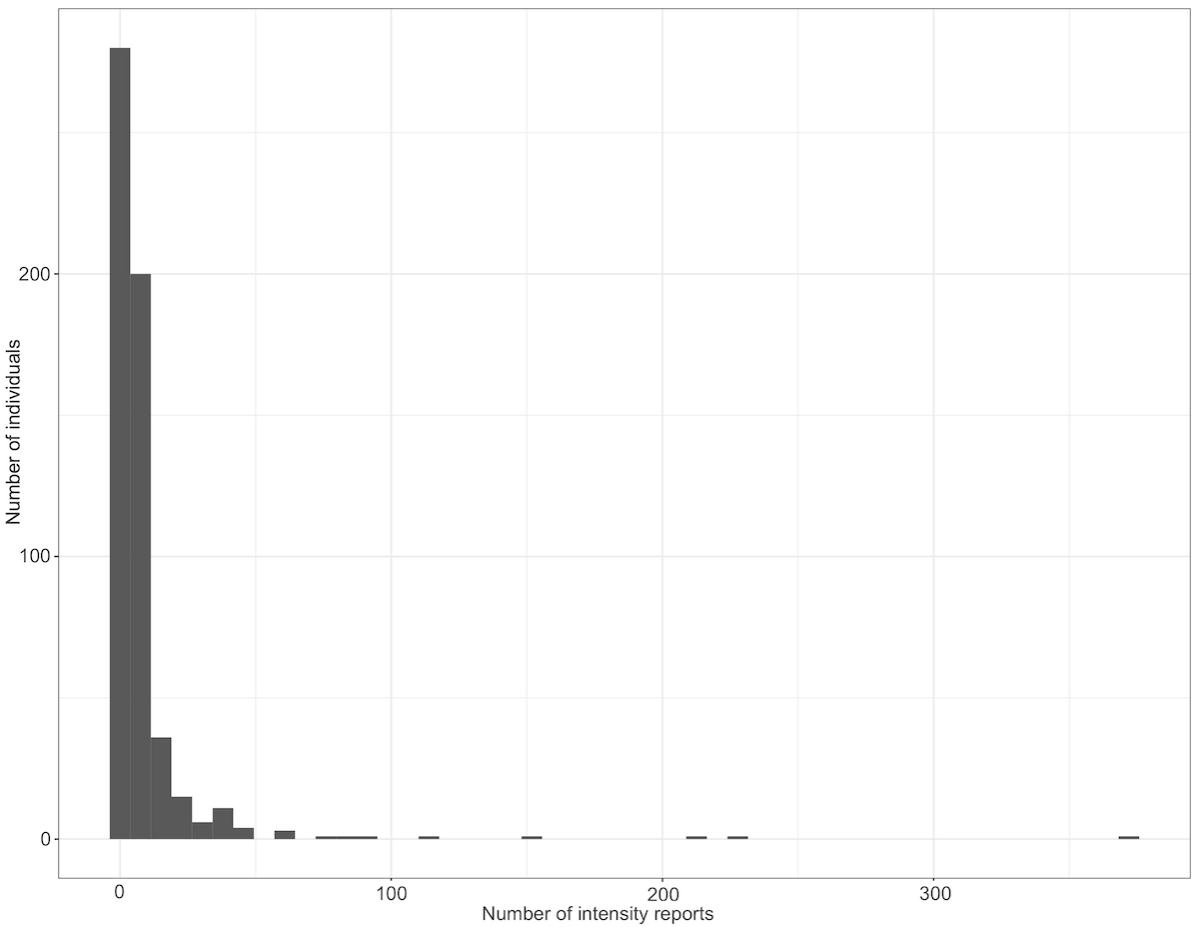
Histogram of the number of intensity reports and number of users among 731 persons newly vaccinated against COVID-19 between July 21, 2021, and September 27, 2022.

There was substantial variance in the frequency with which participants reported their symptoms (see Figure 6).

A total of 563 individuals provided at least one report, with the number of reports ranging from 1 to 373 and a median of 4 (IQR 2-7). If the same symptom was reported multiple times with the same intensity within a 15-minute window, only the first report was considered valid, while subsequent reports were classified as duplicates and removed. By contrast, reports of a different symptom or the same symptom with a different intensity were considered new reports. Using this definition, 434 participants provided multiple symptom reports. The time until the next report was a median of 21 hours, with an IQR of 9-33 hours.

A total of 143 unique symptoms were reported, including synonyms, different spellings, and other free-text inputs. Among these, 29 symptoms were suggested to users based on the most common vaccine side effects or symptoms of COVID-19. Of the 143 unique entries in the symptoms field, 42 were reported only once. The most frequently reported symptom, pain or tenderness at the injection site, was mentioned 1486 times by 486 individuals. Among recurrent users, 153 individuals reported at least one symptom 10 times or more, with the most frequent user documenting symptoms 376 times during the data export period.

**Figure 7:**
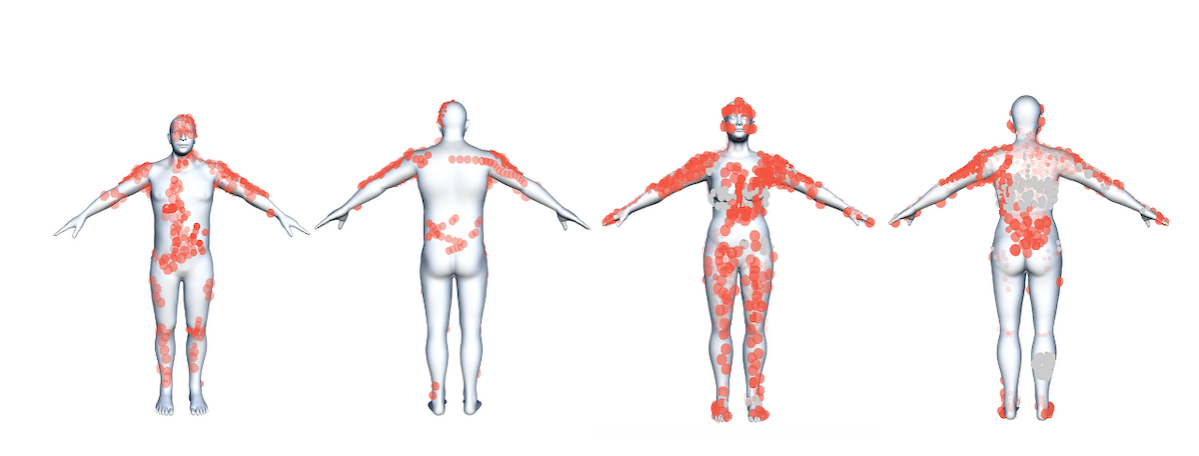
All drawn data (from all individuals during the data export period between July 21, 2021, and September 27, 2022) combined and portrayed on a male (left) and female (right) manikin. Intensity is shown by opacity: the darker the color the higher the intensity. Gray indicates that the intensity of the symptom is set to 0 at the time of data export.

**Figure 8:**
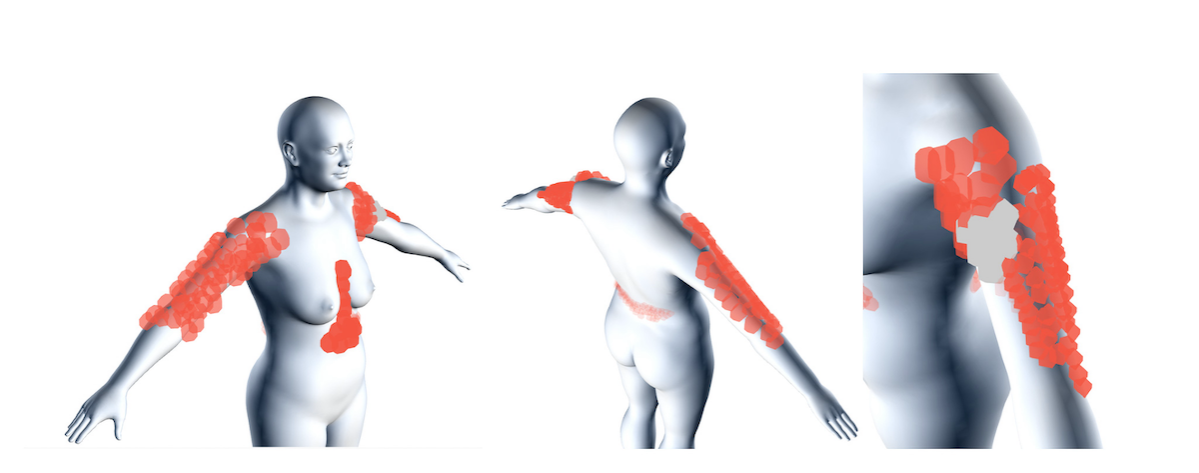
Data reported during the first month of the study (between July 21, 2021, and August 21, 2021) on the female manikin. The rightmost image shows a zoomed in version of the left upper arm.

A total of 33 of the 563 individuals (5.9%) localized their symptoms by drawing them on the 3D manikin, with variations in localization, incidence, and the extent of the drawings. Figure 7 presents collated data from all drawings by different individuals, displayed on both the male and female 3D manikins. Figure 8 shows all data drawn on the female 3D manikin during the first month of the study.

## Discussion

### Use of the Symptoms System

Individuals primarily used the system in connection with vaccinations, that is, shortly after their recruitment alongside vaccination. However, some individuals utilized the system for longer periods. This finding aligns with earlier research highlighting individual differences in preferences for using mHealth technology [31-33]. Symptom drawings were not utilized as frequently as anticipated, despite usability testing and adaptations made to clarify the use of the manikins before the study began. Further adjustments have now been implemented to enhance the use of the 3D manikin. Users are now prompted to draw the localization on the 3D manikin after selecting a symptom to report. In this study, however, participants might not have felt the need to draw symptoms, as the meaning of terms such as “pain at the injection site” is clear, and a drawing may not provide significant added value for the individual. The system serves as a personal health diary, allowing users to record the level of detail they find important. If users had been more clearly informed that their drawings and detailed reports could benefit downstream research projects, they might have been more willing to share data, potentially increasing the number of drawings submitted. Similarly, providing instructions for consistent reporting, such as weekly updates over a longer period, could have yielded more reliable longitudinal data. It is important to note that different methods of eliciting data can influence results. These insights represent valuable lessons for future implementations.

Most symptoms reported were not included in the list of suggested symptoms, underscoring the importance of allowing individuals to determine which symptoms they wish to track. As patients often find it challenging to remember symptom levels, such as the intensity of pain and fatigue, beyond the past few days [10], this further emphasizes the necessity for researchers to collect data at the time symptoms occur, rather than at a later time point. Patients’ descriptions of their symptoms are crucial for treatment decisions and for enabling patients to monitor their own symptoms [2-4,10]. As a result of this study, the *Symptoms* system has been updated to include a wide array of prespecified symptoms based on the SNOMED Clinical Terms (SNOMED CT) ontology. These symptoms appear as suggestions when users enter free text, and measures are also being implemented to learn from the free-text symptoms that users add. The National Board of Health and Welfare in Sweden serves as a national release center for SNOMED CT, collaborating with supporting stakeholders to utilize the ontology and ensure it remains up to date. If many free-text symptoms are added to the *Symptoms* system that do not correspond well to established SNOMED CT concepts, it is possible to suggest additions of concepts or new synonyms to the National Board of Health and Welfare. This approach can enhance the list of prespecified symptoms in the system while ensuring that the data remain valuable for clinical documentation in the future. The latest version of the *Symptoms* system includes a subset of SNOMED CT, allowing terms to appear as suggestions when users begin typing in the field where they add and name symptoms. This feature facilitates data analysis, allowing users to select an established and mapped SNOMED CT concept. However, users still have the option to type their own name or term for the symptom. In addition to incorporating the SNOMED CT symptom or their self-defined symptom, users can now include a text description of the symptom and provide a drawing to illustrate it, if they wish.

As recruitment for the study began after many individuals in the older population had received their first and second COVID-19 vaccine doses, the population reached at the vaccination clinics during the study was relatively young and healthy, with 70.9% (498/702) reporting no listed comorbidities. This may explain why many participants used the system primarily in connection with vaccinations and for a relatively brief period thereafter. As participants were recruited at vaccination sites and asked about current symptoms rather than asked to recall symptoms later, recall bias may be less of a concern compared with other studies where participants reported side effects at a later stage without a direct connection to the vaccination. The use of mobile devices for real-time reporting of side effects likely contributed to accurate rates, as the frequencies of reported symptoms appear to align with previously documented side effects [34-37], as discussed below.

On the 3D manikin, individuals exhibited variation in the localization and expansion of drawn symptoms. However, for both men and women, the arms were common areas, as expected, with “pain at the injection site” being a frequently drawn symptom. Other reported symptoms included “pain,” “stomach pain,” “creaking joints,” “rash,” and “swelling.”

Only a few individuals reached out for support with the self-reporting tool, and the emails primarily contained suggestions, such as requesting a clearer method for reporting multiple COVID-19 tests. This may indicate that the system was relatively self-explanatory, requiring minimal support for effective use.

### Symptoms Reported and Comparisons With Prior Work

The most common symptoms reported in this study were pain at the injection site (486/563, 86.3%), fatigue (181/563, 32.1%), and headache (169/563, 30%). Slightly less common, but still reported by many individuals, were muscle pain (160/563, 28.4%) and fever (148/563, 26.3%). Overall, of the 731 participants, 563 (77%) reported experiencing symptoms.

Previous reports have shown similar findings regarding symptoms following COVID-19 vaccinations. A safety update on COVID-19 vaccines published by the Advisory Committee on Immunization Practices at the U.S. Centers for Disease Control and Prevention reported that local tenderness (74.8%) was the most common side effect, followed by fatigue (50.0%), headache (41.9%), myalgia (41.6%), chills (26.7%), and fever (25.2%) after the second dose of the Comirnaty vaccine [34]. The same symptoms were reported, but less frequently, after the first dose of the same vaccine [34]. Similar findings have been reported in multiple studies [35-37]. The *Symptoms* system appeared to capture symptoms or self-experienced side effects to a similar extent as other studies on vaccine follow-up [34-37]. There are concerns that methods used to elicit participant-reported symptoms can influence the detection of these data [38]. Therefore, it is not unexpected that the exact observed frequencies of different symptoms vary between this study and other studies on vaccine follow-up.

### Strengths and Limitations

Despite the presence of posters and flyers at many vaccination points in Västra Götalandsregionen (VGR), a limited number of individuals chose to participate in the study. A potential selection bias may exist, as recruitment occurred when the older population had already received their initial 2 vaccination shots, and the individuals reached by the advertisements were primarily younger adults. As a result, the study population was relatively young and healthy, which may have influenced symptom reporting. Additionally, the overrepresentation of females (444/731, 60.7%) aligns with previous studies, which have observed that women are more likely to participate in digital health surveys [23,35,36,39].

A strength of the study is the use of a digital application, particularly given the high level of digital competence in Sweden, where 94% of the population uses the internet and almost everyone accesses it daily [40]. Users could utilize the system—a web app—on various devices, including smartphones, tablets, or computers. A potential selection bias may arise from the fact that, in order to obtain a BankID, individuals must have a Swedish PIN and be customers of one of the banks that issue BankID. People who are not residing in Sweden or who are studying in Sweden for less than 1 year cannot obtain a Swedish PIN. Additionally, newcomers to Sweden must wait for the administrative process to obtain a Swedish PIN. However, BankID was used by 97% of internet users in Sweden in 2022 [40].

It is reasonable to assume that there are technical obstacles associated with any technological system. In this study, no metadata or logs were utilized to track whether participants encountered technical problems that hindered their use of the system. Similarly, there is no information on how many individuals saw the posters but were unable to join the study due to technical or other reasons. Among those who created an account, only a few did not complete the survey, and we cannot determine whether noncompletion was due to technical issues or other factors. Additionally, only a small number of participants reached out to the support email with technical questions.

Differences in the use of certain vaccines were anticipated due to the vaccination program and the availability of different vaccines. As the program developed, these factors contributed to a significant majority of individuals in Sweden receiving the Comirnaty vaccine.

The study relied on self-reporting, and symptoms were not verified through any other means. Furthermore, individuals who experienced side effects may have been more motivated to participate and contribute more data and longer follow-ups than those who did not, potentially leading to an overestimation of symptom rates and duration. Therefore, participants using the app system are expected to be a self-selected group that may not fully represent the general population. Additionally, the absence of a control group comprising nonvaccinated individuals limits the ability to conclusively determine the extent to which the self-reported symptoms can be attributed to the vaccination.

The flexibility of the self-directed system, which allows individuals to determine what they consider worth tracking as symptoms, proved to be important, as the prespecified symptoms accounted for only a small portion of the diverse symptoms reported.

### Conclusions

Self-reported symptoms in the *Symptoms* system appeared to align with previously observed experiences following COVID-19 vaccination. The system was generally easy to use and effectively captured a wide range of data in a longitudinal manner. Its person-centered and self-directed design seemed crucial for documenting the full burden of symptoms experienced by users.

## Appendix

Multimedia Appendix 1 Translation of screenshots in Figures 1-4.

Multimedia Appendix 2 A video walk-through of how the system works for the user when they first log in after seeing the posters.

Multimedia Appendix 3 Questionnaire with translation.

## Abbreviations

mHealth: mobile health
NRS: numeric rating scale
PIN: Personal Identity Number/personnummer
PROM: patient-reported outcome measure
RECOVAC: Register-based large-scale national population study to monitor COVID-19 vaccination effectiveness and safety
SCIFI-PEARL: Swedish COVID-19 Investigation for Future Insights, a Population Epidemiology Approach using Register Linkage
SNOMED CT: SNOMED Clinical Terms
VGR: Västra Götalandsregionen

## Data Availability

The data sets generated during and analyzed during this study are available from the corresponding author on reasonable request. The data are not publicly available because they contain information that could compromise the privacy of research participants.
