# Supplementary material for: Person-Centered Web-Based Mobile Health System (Symptoms) for Reporting Symptoms in COVID-19 Vaccinated Individuals: Observational Study of System, Users, and Symptoms
Source: JMIR Form Res. 2024 Oct 30;8:e57514. doi: 10.2196/57514 (PMC11561448; doi:10.2196/57514)
Supplement: Multimedia Appendix 1 [file formative_v8i1e57514_app1.pdf]

Multimedia Appendix 1 – Gustafson Hedov et al. **Person-Centered Web-Based Mobile Health System (Symptoms) for Reporting Symptoms in COVID-19 Vaccinated Individuals: Observational Study of System, Users, and Symptoms.** 2024.

Supplementary material – Translation of screenshots in Figures 1-4.

Translations from Swedish to English are presented in text boxes.

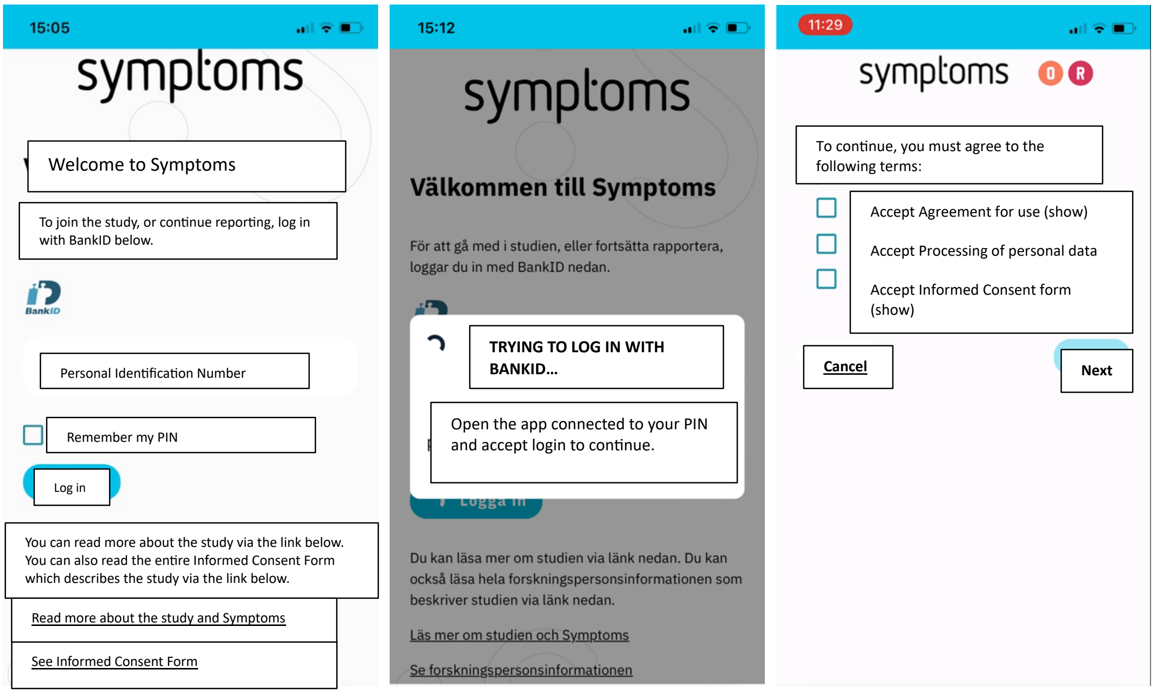

Figure 1. Screenshots from the Symptoms system during recruitment for the study (from July 2021): The landing page, login screen waiting for approval by BankID, and screen for approving agreements.

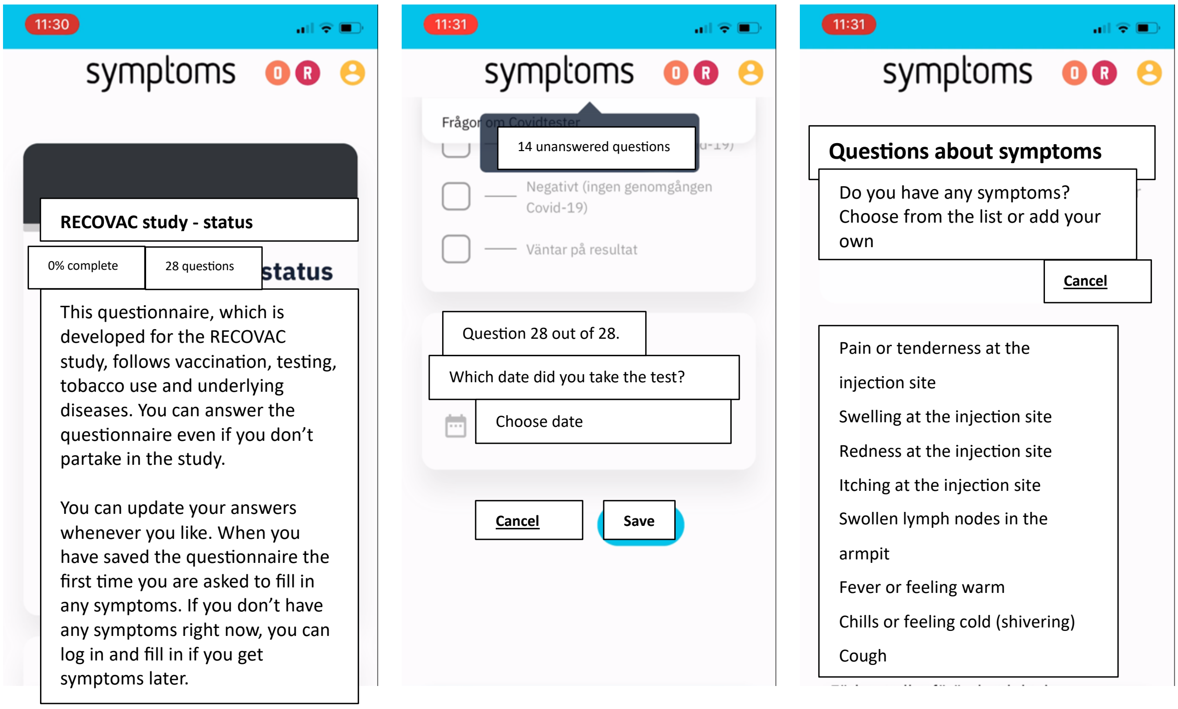

Figure 2. Screenshots from the Symptoms system during the study (from July 2021-Sept 2022): The start and end of the initial questionnaire, and (far right) the subsequent screen for initial symptom reporting, where common symptoms or side effects are listed. The users were also free to type their own symptoms.

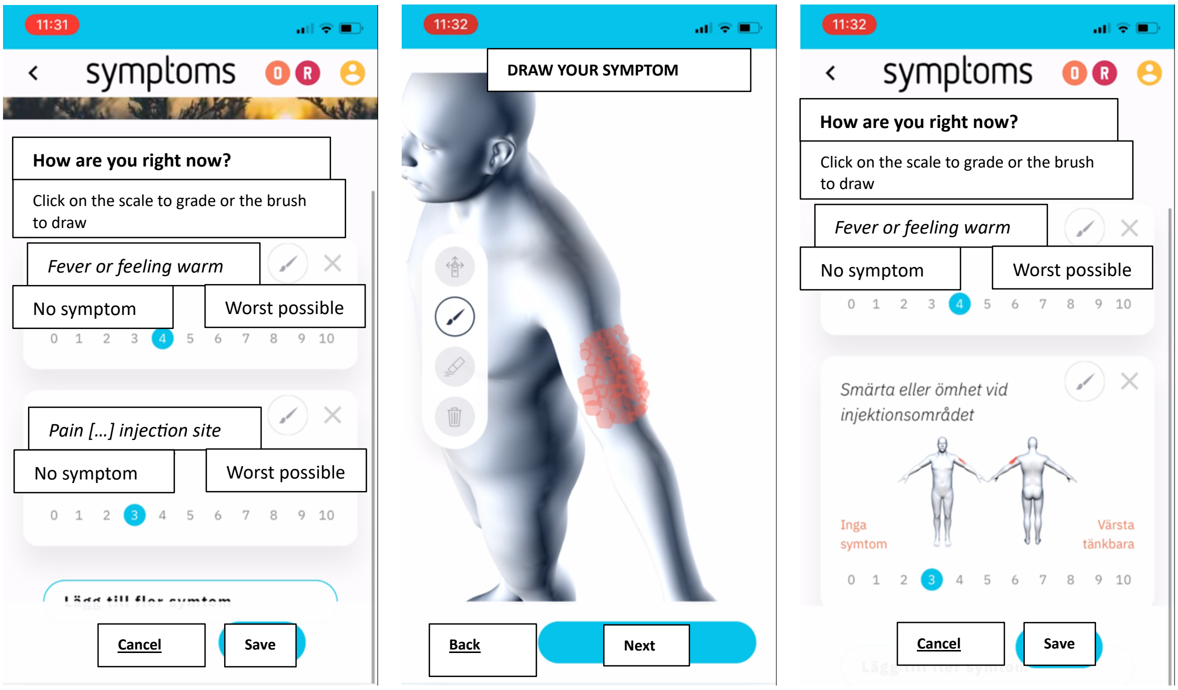

Figure 3. Screenshots from the Symptoms system during the study period (July 2021 to Sept 2022): Grading of symptoms, drawing of symptoms on the 3D-manikin and grading of symptoms with updated drawing.

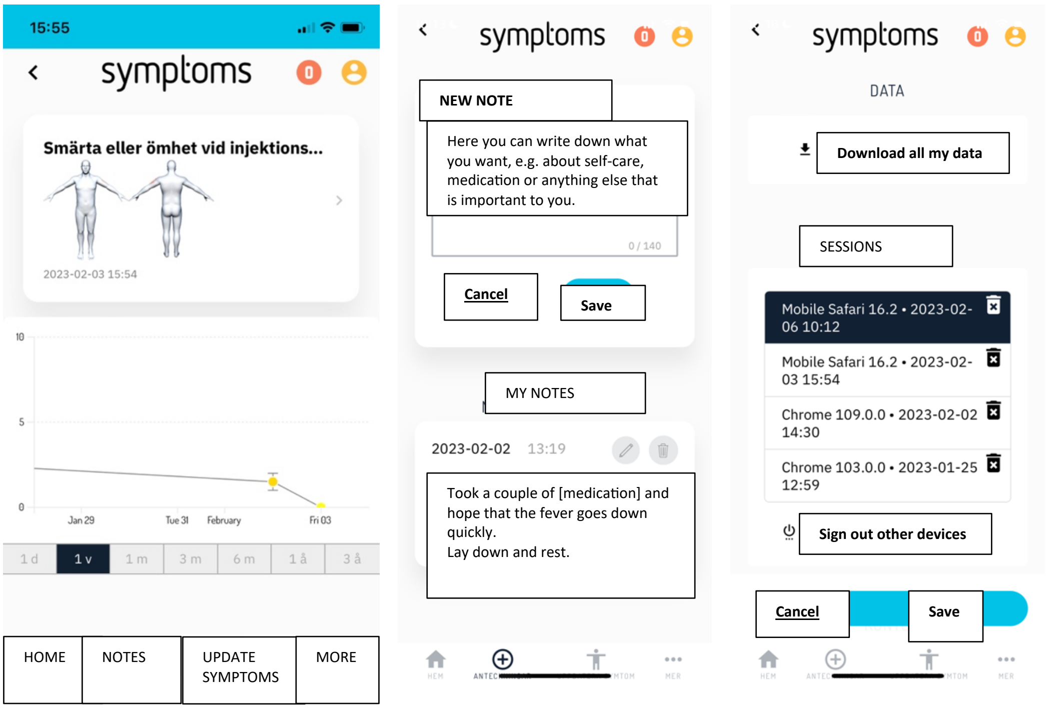

Figure 4. Screenshots from the Symptoms system as it looked during the study period (July 2021 to Sept 2022): Graph of a symptom with a corresponding drawing, screen for free-text notes, and settings page with options for data export of a person's own Symptoms data, and active sessions on the same or different devices.
