# Supplementary material for: Person-Centered Web-Based Mobile Health System (Symptoms) for Reporting Symptoms in COVID-19 Vaccinated Individuals: Observational Study of System, Users, and Symptoms
Source: JMIR Form Res. 2024 Oct 30;8:e57514. doi: 10.2196/57514 (PMC11561448; doi:10.2196/57514)
Supplement: Multimedia Appendix 3 [file formative_v8i1e57514_app3.pdf]

Supplementary material – Questionnaire

Answer options are given in angle brackets. Some questions are only shown if the previous question is answered affirmatively, such as for smoking and snuff (this is indicated by [If yes] in the questionnaire).

Denna enkät, som är framtagen för RECOVAC-studien, följer upp vaccinering, provtagning, tobaksvanor och bakomliggande sjukdomar. Du kan fylla i enkäten även om du inte deltar i studien.<br><br>Du kan uppdatera dina svar när du vill. När du sparat enkäten den första gången ombeds du fylla i dina eventuella symtom. Har du inga symtom just nu kan du logga in och fylla i om du får symtom senare.	This questionnaire, which is developed for the RECOVAC study, asks about vaccination, testing, tobacco use and underlying diseases. You can answer the questionnaire even if you don't partake in the study.<br><br>You can update your answers whenever you like. When you have saved the questionnaire the first time you are asked to fill in any symptoms you have. If you don't have any symptoms right now, you can log in and fill in if you get symptoms later.
Bakgrundsinformation	Background information
1. Längd (cm) [siffra 100-250]<br><br>2. Vikt (kg) [siffra 30-299]<br><br>3. Är du eller du har varit rökare? [ja/nej]<br><br>[Om ja]<br>4. Hur många år har du rökt sammanlagt [dropdown med siffror 1-100]?<br><br>5. Hur många cigaretter om dagen har du i genomsnitt rökt under din tid som rökare? [dropdown med siffror 1-20; 1 paket; 1,5 paket; 2 paket; 2 eller fler paket]<br><br>6. Om du slutat röka, vid vilken ålder slutade du? [dropdown med siffror 1-100; Har inte slutat]<br><br>7. Om du röker, hur många cigarretter om dagen röker du nu? [dropdown med siffror 1-20; 1 paket; 1,5 paket; 2 paket; 2 eller fler paket]	Height (cm) [number 100-250]<br><br>Weight (kg) [number 30-299]<br><br>Are you or have you been a smoker? [yes/no]<br><br>[If yes]<br>How many years have you smoked in total? [dropdown with numbers 1-100]<br><br>How many cigarettes a day have you smoked on average during your time as a smoker? [dropdown with numbers 1-20; 1 pack; 1.5 pack; 2 pack; 2 or more packs]<br><br>If you quit smoking, at what age did you quit? [dropdown med numbers 1-100; Haven't stopped]<br><br>If you smoke, how many cigarettes a day do you smoke now? [dropdown with numbers 1-20; 1 pack; 1.5 pack; 2 pack; 2 or more packs]

8. Är du eller har du varit snusare? [ja/nej]<br><br>[Om ja]<br>9. Hur många år har du snusat sammanlagt [dropdown med siffror 1-100]?<br><br>10. Hur många dagar har en dosa snus räckt under din tid som snusare? [dropdown med siffror 1-14]<br><br>11. Om du slutat snusa, vid vilken ålder slutade du? [dropdown med siffror 1-100]<br><br>12. Om du snusar, hur många dagar räcker en dosa snus nu? [dropdown med siffror 1-14]<br><br>13. Har du eller har du haft någon av följande sjukdomar [multiple choice:<br>□ Hjärtinfarkt/propp i hjärtat<br>□ Kärlkramp/angina pectoris<br>□ Förmaksflimmer<br>□ Hjärtsvikt<br>□ Klaffel på hjärtat<br>□ Stroke/slaganfall/hjärninfarkt/propp i hjärnan/hjärnblödning<br>□ Högt blodtryck<br>□ Höga blodfetter/högt kolesterol<br>□ Diabetes/sockersjuka<br>□ Kronisk obstruktiv lungsjukdom (KOL), kronisk bronkit eller emfysem<br>□ Astma<br>□ Annan lungsjukdom (annat än kronisk obstruktiv lungsjukdom (KOL), kronisk bronkit eller emfysem)<br>□ Morbus Crohn eller ulcerös kolit<br>□ Reumatisk sjukdom, tex reumatoid artrit/Bechterews sjukdom/psoriasisartrit/SLE/Sjögrens syndrom<br>□ Cancer<br>□ Inga av ovanstående sjukdomar]	Are you or have you been a snuffer? [yes/no]<br><br>[If yes]<br>How many years have you used snuff in total [dropdown with numbers 1-100]?<br><br>How many days has a box of snuff lasted you during your time as a snuffer? [dropdown with numbers 1-14]<br><br>If you stopped using snuff, at what age did you stop? [dropdown with numbers 1-100]<br><br>If you use snuff, how many days does a box of snuff last now? [dropdown with numbers 1-14]<br><br>Do you have or have you had any of the following conditions [multiple choice:<br>□ Myocardial infarction/clot in the heart<br>□ Angina pectoris<br>□ Atrial fibrillation<br>□ Heart failure<br>□ Heart valve failure<br>□ Stroke/stroke/cerebral infarction/clot in the brain/brain haemorrhage<br>□ High blood pressure<br>□ High blood fats/high cholesterol<br>□ Diabetes<br>□ Chronic obstructive pulmonary disease (COPD), chronic bronchitis or emphysema<br>□ Asthma<br>□ Other lung disease (other than chronic obstructive pulmonary disease (COPD), chronic bronchitis or emphysema)<br>□ Crohn's disease or ulcerative colitis<br>□ Rheumatic disease, e.g. rheumatoid arthritis/Bechterew's disease/psoriatic arthritis/SLE/Sjögren's syndrome<br>□ Cancer<br>□ None of the above conditions]
Frågor om vaccinering	Questions about vaccination

14. Jag har fått första dosen vaccin mot Covid-19 [yes/no]<br><br>[Om ja]<br>15. Vilket vaccin fick du? - [Astra Zeneca; Pfizer; Moderna; Annat]<br><br>16. Vilket datum? [datum]<br><br>17. Jag har fått andra dosen vaccin mot Covid-19 [checkbox]<br><br>[Om ja]<br>18. Vilket vaccin fick du? - [Astra Zeneca; Pfizer; Moderna; Annat]<br><br>19. Vilket datum? - [datum]	I have received the first dose of vaccine against Covid-19 [yes/no]<br><br>[If yes]<br>Which vaccine did you receive? - [Astra Zeneca; Pfizer; Moderna; Other]<br><br>What date? [starting point]<br><br>I have received the second dose of vaccine against Covid-19 [check box]<br><br>[If yes]<br>Which vaccine did you receive? - [Astra Zeneca; Pfizer; Moderna; Other]<br><br>What date? [date]
Frågor om Covidtester<br><br>20. Jag har testat mig för pågående Covid-19 (PCR) [ja/nej]<br><br>[Om ja]<br>21. Vad var testresultatet? [Positivt (pågende Covid-19); Negativt (ingen pågående Covid-19; Väntar på resultat)<br><br>22. Vilket datum gjorde du testet? [datum]<br><br>23. Jag har testat mig för genomgången Covid-19 (antikroppar) [ja/nej]<br><br>[Om ja]<br>24. Vad var testresultatet? [Positivt (genomgången Covid-19); Negativt (ingen genomgången Covid-19; Väntar på resultat)]<br><br>25. Vilket datum gjorde du testet? [datum]	Questions about Covid tests<br><br>I have been tested for ongoing Covid-19 (PCR) [yes/no]<br><br>[If yes]<br>What was the test result? [Positive (ongoing Covid-19); Negative (no ongoing Covid-19; Awaiting results)<br><br>What date did you take the test? [date]<br><br>I have been tested for past Covid-19 infection (antibodies) [yes/no]<br><br>[If yes]<br>What was the test result? [Positive (past Covid-19); Negative (no past Covid-19; Awaiting results)]<br><br>What date did you take the test? [date]
